# 基于响应面法的基质固相分散萃取土壤中有机磷阻燃剂

**DOI:** 10.3724/SP.J.1123.2023.04018

**Published:** 2024-01-08

**Authors:** Junxia WANG, Sijie XU, Yueying SUN, Huihui LEI, Yuanyuan CHENG, Xuedong WANG, Zhan’en ZHANG

**Affiliations:** 1.苏州科技大学环境科学与工程学院, 江苏 苏州 215009; 1. School of Environmental Science and Engineering, Suzhou University of Science and Technology, Suzhou 215009, China; 2.苏州科技大学江苏省环境科学与工程重点实验室, 江苏 苏州 215009; 2. Jiangsu Key Laboratory for Environmental Science and Engineering, Suzhou University of Science and Technology, Suzhou 215009, China

**Keywords:** 气相色谱-串联质谱, 基质固相分散萃取, 响应面法, 有机磷阻燃剂, 土壤, gas chromatography-tandem mass spectrometry (GC-MS/MS), matrix solid-phase dispersion extraction (MSPD), response surface methodology (RSM), organophosphorus flame retardants (OPFRs), soil

## Abstract

有机磷阻燃剂(OPFRs)被广泛添加于商业品和日用品中,由于具有环境持久性、生物富集性和潜在毒性,已成为一种新兴的持久性有机污染物。因此需要建立能准确定量环境中OPFRs的检测方法。该文基于基质固相分散萃取(MSPD),结合气相色谱-串联质谱(GC-MS/MS)法测定土壤中10种有机磷阻燃剂,筛选对OPFRs具有高选择性的吸附剂,最终确定MSPD最佳萃取条件。该文基于单因素分析法考察了常见吸附剂(C18、PSA、Florisil、石墨化炭黑(GCB)和多壁碳纳米管(MWCNT))及其用量、洗脱溶剂及其体积、研磨时间对OPFRs萃取效率的影响。在此结果基础上,进一步利用响应面法(RSM)考察了3个关键因素(吸附剂用量、洗脱剂用量和研磨时间)以及交互作用对OPFRs萃取效率的影响。最终确定最优条件:吸附剂GCB,用量0.3 g;洗脱溶剂乙酸乙酯,用量10 mL;研磨时间5 min,此时10种OPFR的萃取效率为87.5%。在GC-MS/MS的多反应监测模式(MRM)下,以^13^C-PCB208为内标物进行定量。10种OPFRs在6个浓度梯度下,获得较好的线性,相关系数大于0.998。该方法的LOD和LOQ分别为0.006~0.161 ng/g和0.020~0.531 ng/g。在最佳条件下,加标土壤中OPFRs的加标回收率为70.4%~115.4%,相对标准偏差(RSD)为0.7%~6.7%。将该方法用于苏州不同功能土壤中OPFRs的含量测定,结果表明电子厂和汽修厂土壤中OPFRs总含量显著高于稻田土和校园土,电子厂和汽修厂土壤中主要污染物为磷酸三(2-氯异丙基)酯(TCIPP)、三苯基氧化膦(TPPO)、磷酸三(2-氯乙基)酯(TCEP)和磷酸三(1,3-二氯-2-丙基)酯(TDCPP),它们在电子厂土壤中含量分别为5.30、4.44、4.54、4.20 ng/g,在汽修厂土壤中的含量分别2.70、3.93、7.60、5.04 ng/g。目前关于TPPO的土壤污染报道较少,该研究在苏州工业区土壤中检出了高浓度TPPO污染。该方法成功用于土壤中10种OPFRs的检测。

## Abstract (en)

Organophosphorus flame retardants (OPFRs) are widely used in commercial products owing to their exceptional flame-retarding and plasticizing properties. However, OPFRs are also well recognized as emerging persistent organic pollutants (POPs) because of their environmental persistence, biological concentration, and potential toxicity. Thus, the accurate detection of OPFRs in environmental media is critical for analyzing their fate, transport, and ecological risk. However, very few OPFR detection methods are currently available, and the types of OPFRs detected may vary from site to site.

In this study, matrix solid-phase dispersion extraction (MSPD), a simple, rapid, and versatile technique for preparing solid, semisolid, liquid, and viscous samples, was combined for the first time with gas chromatography-tandem mass spectrometry (GC-MS/MS) to analyze 10 OPFRs in soil, namely, tripropyl phosphate (TPrP), tri-*n*-butyl phosphate (TnBP), tri-iso-butyl phosphate (TiBP), tris(2-chloroisopropyl) phosphate (TCIPP), tris(2-chloroethyl) phosphate (TCEP), tris(1,3-dichloro-2-propyl) phosphate (TDCPP), triphenyl phosphate (TPHP), 2-ethylhexyl diphenyl phosphate (EHDPP), triphenylphosphine oxide (TPPO), and trimethylphenyl phosphate (TCP). The GC-MS/MS system was equipped with a Bruker-5MS capillary column coupled with a triple quadrupole mass spectrometer operated in multiple reaction monitoring (MRM) mode. Prior to detection, a mixed standard solution was fortified with 10 ng of^13^C-PCB208 as an internal standard. The optimal conditions under which MSPD could achieve high selectivity for OPFRs were determined. In addition, single-factor analysis was used to examine the influence of the sorbent (i. e., C18, PSA, Florisil, GCB, and multiwalled carbon nanotubes (MWCNTs)) as well as the dosage, type, and volume of the eluent on the extraction efficiency of the method for the 10 OPFRs. When GCB and ethyl acetate were used as the adsorbent and solvent, respectively, during elution, high extraction recoveries for the OPFRs were achieved. Optimization via response surface methodology (RSM) was adopted to further analyze the impact of three key factors, namely, the adsorbent dosage, eluent volume, and grinding time, as well as their interactions, on OPFR recoveries. Under the optimal conditions of 0.3 g of GCB as the adsorbent, 10 mL of ethyl acetate as the eluent, and 5 min of grinding time, the relative average recovery of the OPFRs was 87.5%. Furthermore, the 10 OPFRs showed good linear relationships under five concentration gradients, with correlation coefficients greater than 0.998. The limits of detection (LODs) and quantification (LOQs) were calculated as signal-to-noise ratios (*S/N*) of 3 and 10, respectively, and found to be in the ranges of 0.006-0.161 and 0.020-0.531 ng/g, respectively. The performance of the proposed method was verified by determining the recoveries and relative standard deviations (RSDs) of the OPFRs in soils spiked at low, medium, and high levels (10, 20, and 100 ng/g, respectively). The recoveries of the OPFRs ranged from 70.4% to 115.4%, with RSDs ranging from 0.7% to 6.7%. Compared with the conventional accelerated solvent extraction (ASE) method, MSPD presents higher efficiency, simpler operation, and less solvent requirements. The developed method was applied to determine OPFRs in soil samples collected from different sites in Suzhou, including an electronics factory, an auto-repair factory, a paddy field, and a school field. The results revealed that the contents of OPFRs in the soils from the electronics and auto-repair factories were significantly higher than those in the soils from the paddy and school fields. The main pollutants in the soil samples collected from the electronics and auto-repair factories were TCIPP, TPPO, TCEP, and TDCPP. Moreover, the contents of these compounds were 5.30, 4.44, 4.54, and 4.20 ng/g, in soils from the electronics factory and 2.70, 3.93, 7.60, and 5.04 ng/g, in soils from the auto-repair factory. To the best of our knowledge, this study is the first to determine high concentrations of TPPO in industrial soils. Thus, the combination of MSPD and GC-MS/MS adopted in this study can provide useful insights into the detection of the 10 OPFRs in soil.

随着溴化阻燃剂(BFRs)在全球范围内先后被管制与禁用,有机磷阻燃剂(organophosphorus flame retardants, OPFRs)作为主要的替代品之一,被广泛添加到各种纺织品、家具以及电子产品中^[1]^。OPFRs具有环境持久性以及远距离运输潜力^[2]^,导致其易于进入各种环境介质中,在水体^[3]^、大气^[4]^、灰尘^[5]^、沉积物^[6]^和土壤^[7]^中均已发现OPFRs的存在,尤其已在多种废物回收区^[8]^、电子废物回收场地周围的土壤和灰尘^[9]^中检测到高浓度OPFRs。大量的动物实验和流行病学研究证明OPFRs对人类与动物存在不利影响,一些OPFRs对生物体具有显著的内分泌干扰毒性和代谢毒性^[10]^。土壤是OPFRs的污染汇之一,准确测定土壤中OPFRs的含量,将为其环境行为和生态系统风险研究提供基础。

对于复杂固体基质样品中OPFRs的前处理往往包括提取和净化两个步骤,一般用超声辅助提取或加速溶剂萃取(ASE)^[11]^先进行提取,后用复合硅胶柱或Florisil柱净化^[12]^,前处理时间约为2~5 h。为了实现土壤样品中OPFRs污染物的准确、可靠、快速定量,选择合适的样品前处理技术至关重要。基质固相分散萃取(matrix solid-phase dispersion extraction, MSPD)是一种集提取、净化于一体,高效且简单的提取方法^[13]^,已被用作灰尘样品中BFRs的提取和净化^[14]^,通常与液相色谱(LC)^[15]^和气相色谱(GC)^[16]^联合使用。MSPD的关键在于吸附剂,常见的吸附剂有键合硅胶C18^[17]^、极性吸附材料Florisil^[18]^、极性键合相*N*-丙基乙二胺(PSA)^[19]^、多壁碳纳米管(MWCNT)^[20]^和石墨化炭黑(GCB)^[21]^等。对OPFRs选择性越高的吸附剂,方法的灵敏度越高,因此需筛选出最优吸附剂。吸附剂用量、洗脱溶剂及其体积影响MSPD萃取,因此需精心优化MSPD萃取参数,以期达到最佳的萃取效果。传统单因素分析的步骤是改变一个变量,其他参数保持不变,但这种方法缺少变量之间相互作用的信息,多个变量需要试验批次较多,试剂耗量大^[22,23]^,因此需寻找一种试验批次较少、能反映变量之间相互作用的分析方法。

响应面法(response surface methodology, RSM)是一种基于多元非线性模型的统计技术,它不仅显示了每个因素(变量)对响应的影响,还能确定这种影响如何随着其他相关因素的变化而变化^[24,25]^。通过有限的试验次数在短时间内找到最佳条件。在响应面法中,中心复合试验设计(central composite design, CCD)适用于多因素、多水平、存在连续变量的试验设计,为待评估的响应提供可靠的试验,之后利用这些试验结果,建立与试验数据最拟合的数学模型,确定最佳实验条件。该文基于RSM优化MSPD的关键影响因素,并结合气相色谱-串联质谱技术(GC-MS/MS)对不同功能区土壤中10种OPFRs进行检测。

## 1 实验部分

### 1.1 仪器、试剂与材料

456GC-SCION TQ气相色谱-三重四极杆质谱联用仪(德国Bruker公司); ASE-350快速溶剂萃取仪(美国Thermo Fisher公司); 12位固相萃取装置(美国Supelco公司); FD-1A-50冻干机(北京博医康); TTL-DCⅠ型氮吹仪(上海Anpel公司); IQ7000 Milli-Q超纯水系统(德国Merck公司)。

C18、PSA、Florisil、GCB和MWCNT均购自美国Sigma-Aldrich公司。乙腈、甲醇均为色谱纯,中性氧化铝和无水硫酸钠为分析纯,均购自上海梯希爱(TCI)化成工业发展有限公司,无水硫酸钠使用前,在马弗炉中450 ℃烘8 h。正己烷(*n*-hexane)、二氯甲烷(DCM)、丙酮(AC)和乙酸乙酯均为分析纯,均购自上海Anpel公司;血清级聚丙烯柱管(6 mL)和筛板购自深圳Biocomma生物科技公司;有机尼龙滤膜(0.22 μm)购自天津津腾实验设备有限公司。

10种有机磷阻燃剂标准品(纯度>98%)包括磷酸三丙酯(TPrP)、磷酸三异丁酯(TiBP)、磷酸三正丁酯(TBP)、磷酸三(2-氯乙基)酯(TCEP)、磷酸三(2-氯异丙基)酯(TCIPP)、磷酸三(1,3-二氯-2-丙基)酯(TDCPP)、磷酸三苯酯(TPHP)、2-乙基己基磷酸二苯酯(EHDPP)、三苯基氧化膦(TPPO)和磷酸三甲苯酯(TCP)均购自上海阿拉丁生化科技股份有限公司。进样内标为^13^C-2,2',3,3',4,5,5',6,6'-九氯联苯(^13^C-PCB208),替代物为氘代磷酸三丁酯(D_27_-TBP),均购自英国剑桥同位素实验室。

准确称取10种OPFRs标准品,用乙腈配制成1000 mg/L的混合标准溶液,于-20 ℃冰箱冷藏保存。使用时用乙腈逐级稀释至适当浓度。

### 1.2 样品前处理

将采集的土壤样品剔除腐烂木屑、石块等杂质,于-20 ℃冰箱中冷冻至少24 h,然后置于冻干机中冷冻干燥24 h,之后研磨,过100目不锈钢筛。精确称取0.20 g土壤样品,加入0.30 g GCB,混合均匀,室温下充分研磨5 min。将研磨后混合物装填进上下带有筛板的6 cm血清级聚丙烯柱管,装填匀实。利用10 mL乙酸乙酯缓慢洗脱,以负压抽真空方式完全收集洗脱液,之后氮吹仪浓缩至尽干,用乙腈复溶至500.0 μL,之后用0.22 μm有机尼龙滤膜过滤,将其保存在2 mL棕色进样瓶中,待进行GC-MS/MS分析。

### 1.3 色谱条件

色谱柱为BR-5MS毛细管色谱柱(15 m×0.25 mm×0.5 μm, Bruker,德国),高纯度氦气(纯度99.999%)作为载气,进样口温度为270 ℃,不分流模式进样,进样体积为2.0 μL;柱流量恒定为1.2 mL/min;升温程序:初始温度90 ℃保持1 min,以25 ℃/min升温至230 ℃,保持1 min,再以15 ℃/min升温至250 ℃,保持2 min。

### 1.4 质谱条件

离子源:电子轰击(EI)电离源,离子源和传输线温度分别为260 ℃和280 ℃,电子能量为-20 eV,碰撞压力为0.266 Pa;选择多反应监测模式(MRM),溶剂延迟时间为3 min。10种有机磷阻燃剂、内标和替代物的离子对和碰撞能量见表1。

**表1:** 10种有机磷阻燃剂、内标和替代物的保留时间、离子对和碰撞能量

Compound	Retention time/min	Ion pairs (m/z)	Collision energy/eV
Tripropyl phosphate (TPrP)	2.99	99.0/81.6, 99.0/99.0^*^	10
Tri-iso-butyl phosphate (TiBP)	3.56	99.0/81.6, 99.0/99.0^*^	10
Tri-n-butyl phosphate (TBP)	4.12	99.0/81.6, 99.0/99.0^*^	10
Tris(2-chloroethyl) phosphate (TCEP)	4.53	249.0/125.3, 205.0/143.0^*^, 99.0/99.0	10
Tris(2-chloroisopropyl) phosphate (TCIPP)	4.65	99.0/81.6, 99.0/99.0^*^, 125.0/125.0	20
Tris(1,3-dichloro-2-propyl) phosphate (TDCPP)	6.90	191.0/77.0^*^, 77.0/51.6, 75.0/75.0	10
Triphenyl phosphate (TPHP)	7.40	170.0/170.0, 170.0/141.5, 325.0/170.0^*^	20
2-Ethylhexyl diphenyl phosphate (EHDPP)	7.60	251.0/251.0^*^, 251.0/153.0, 94.0/66.6	20
Triphenyl phosphine oxide (TPPO)	8.40	277.0/199.0^*^, 199.0/152.0, 77.0/51.0	30
Trimethylphenyl phosphate (TCP)	10.20	165.0/165.0, 368.0/108.0, 368.0/199.0^*^	20
^13^C-Nona-chlorinated biphenyls (^13^C-PCB208)	9.59	476.0/405.9, 478.0/405.9^*^, 403.9/333.6	30
D_27_-Tri-n-butyl phosphate (D_27_-TBP)	4.05	167.0/103.0^*^, 103.0/102.0, 103.0/83.0	10

* Quantitative ion pair.

## 2 结果与讨论

### 2.1 MSPD条件考察

利用单因素法,分别考察了吸附剂种类及其用量、洗脱液种类及其体积和研磨时间对土壤中OPFRs萃取效率的影响,确定主要的、变量连续的影响因素,作为后续响应面法试验设计的变量。

#### 2.1.1 吸附剂

该文选择的10种OPFRs的极性跨度极大,既有极性的TPrP,又有非极性的TCP和EHDPP,因此选择了C18、PSA、Florisil、GCB和MWCNT 5种极性不同的吸附剂,其对土壤中OPFRs进行基质固相分散萃取的结果见图1a。C18与PSA对10种OPFRs的萃取效率较低,可能由于C18主要吸附疏水性强的非极性化合物,而PSA主要吸附极性化合物。Florisil对TPrP、TiBP、TnBP和TDCPP的萃取效率较高,但对其他OPFRs的萃取效率较低。GCB带有芳香性的正六元环结构,呈正电性,具备保留非极性化合物和极性化合物的双重作用。GCB对TPrP、TCEP以及TCIPP的萃取效率高于MWCNT,对TiBP、TBP、EHDPP和TCP萃取效率也稍高于MWCNT,但对TDCPP稍低。当GCB作为吸附剂时,收集的洗脱溶液颜色较MWCNT更无色透明(见附图1, https://www.chrom-China.com),对后续仪器分析的干扰更小。综合考虑,选择GCB作为MSPD的吸附剂。

**图1:**
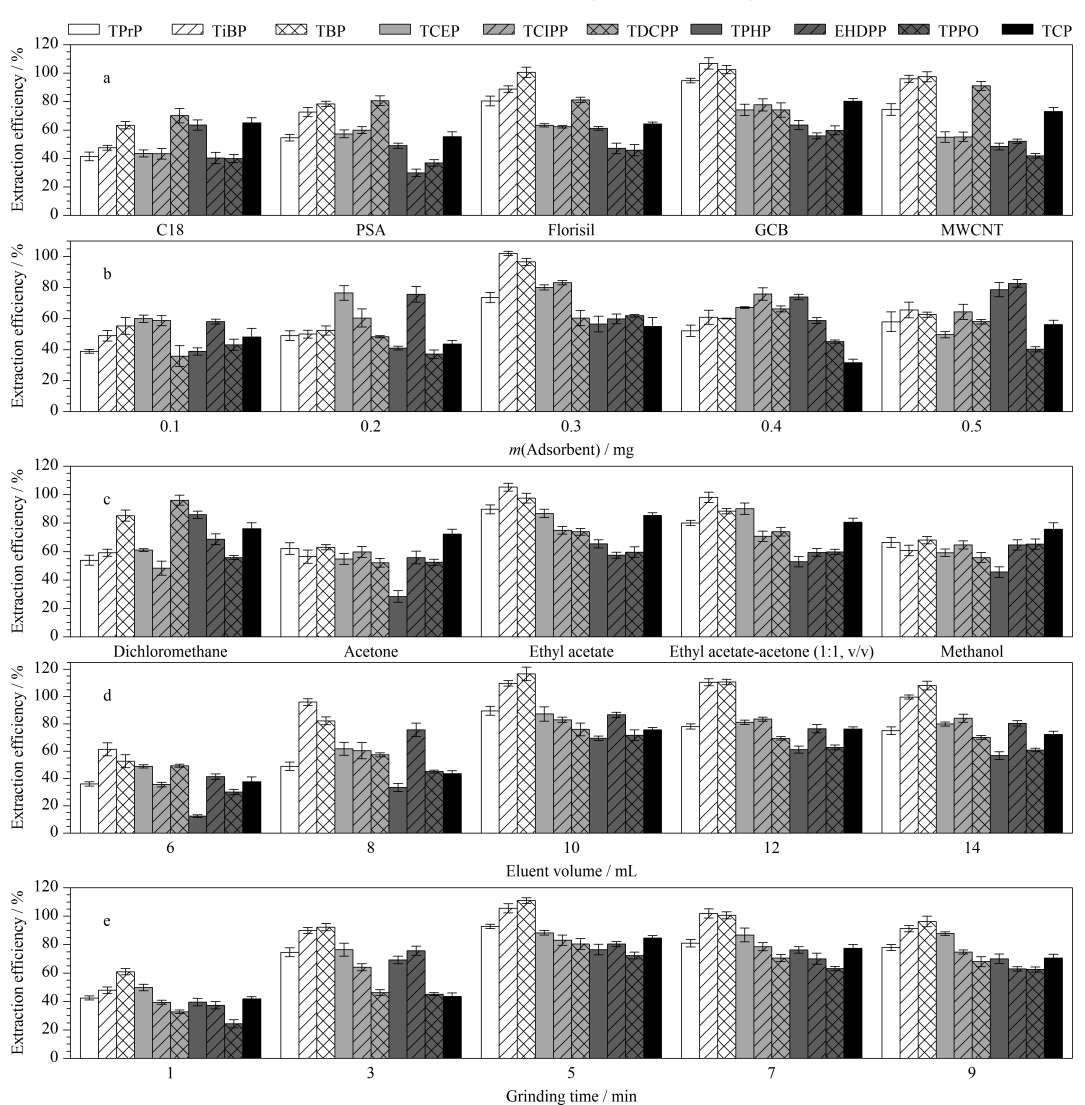
(a)吸附剂、(b)吸附剂用量、(c)洗脱溶剂、(d)洗脱剂体积、(e)研磨时间对10种OPFRs萃取效率的影响(*n*=3)

#### 2.1.2 吸附剂用量

吸附剂用量过少会导致目标物不能被完全吸附,用量过多导致吸附较多干扰物质,增加后续净化步骤,因此需要对吸附剂用量(0.1、0.2、0.3、0.4、0.5 g)进行考察。从图1b可以看出,随着吸附剂用量的增加,各个OPFRs的萃取效率先逐渐增加,在用量达到0.3 g时,TPrP、TiBP、TBP、TCEP、TCIPP和TPPO的萃取效率均达到最大,之后各个OPFRs的萃取效率急剧下降,可能过量的吸附剂从土壤基质中吸附较多OPFRs的同时也吸附更多的干扰物质,导致洗脱困难,使得洗脱下来的目标物数量较少。吸附剂用量为0.3 g时,3种苯基OPFRs(TPHP、EHDPP和TCP)的萃取效率并不是最佳的,尤其是TPHP,其萃取效率在0.5 g时才达到最佳。在萃取效率较高的情况下,尽量选择较少的吸附剂用量,以减少对环境的影响。因此综合考虑,选择吸附剂用量为0.3 g。

#### 2.1.3 洗脱溶剂及其体积

选择洗脱溶剂的依据是“相似相溶”原理,常见的甲醇、丙酮、乙酸乙酯和二氯甲烷等有机溶剂的极性顺序为甲醇>丙酮>乙酸乙酯>二氯甲烷。它们对土壤中10种OPFRs的萃取效率见图1c,洗脱溶剂为极性较强的甲醇和丙酮时,几乎所有OPFRs化合物的萃取效率均较低,洗脱溶剂为中等极性的二氯甲烷时,强疏水性的TDCPP、TPHP和EHDPP的萃取效率达到最大,显著高于其他洗脱溶剂。洗脱溶剂为弱极性的乙酸乙酯时,亲水性强的TPrP、TBP、TiBP、TCEP、TCIPP和TPPO的萃取效率达到最大。同时考察了乙酸乙酯与丙酮的混合溶液(1∶1, v/v),其对强亲水性OPFRs的萃取效率并未显著提高,因此选择乙酸乙酯为洗脱溶剂。

洗脱溶剂体积分别为6、8、10、12和14 mL时OPFRs的萃取效率见图1d。乙酸乙酯用量从6 mL增加到10 mL,各个OPFRs的萃取效率显著增加,在10 mL时达到最佳,继续增加洗脱体积至14 mL, OPFRs的萃取效率并未明显提高,还出现略微下降,这说明10 mL乙酸乙酯几乎完全洗脱吸附在GCB上的OPFRs,因此最终选择洗脱溶剂体积为10 mL。

#### 2.1.4 研磨时间

研磨能够增加基质与吸附剂的接触面积,从而大大增加萃取效率,研磨时间分别为1、3、5、7和9 min时OPFRs的萃取效率见图1e。研磨时间从1 min增加到5 min,所有OPFRs的萃取效率均明显提高,且在5 min时,各个OPFRs的萃取效率达到最大,进一步增加研磨时间至9 min, OPFRs的萃取效率并未继续提高反而有所下降,这可能是由于过度研磨,导致目标物与吸附剂之间吸附亲和力更强,难以将吸附的全部目标物洗脱出来,这与已有研究^[26]^相似。因此最终选择研磨时间为5 min。

### 2.2 中心复合试验设计及结果分析

#### 2.2.1 试验设计与结果分析

基于MSPD的单因子优化结果,以OPFRs化合物的平均萃取效率为响应值*Y*,以*A*(吸附剂用量)、*B*(洗脱剂体积)和*C*(研磨时间)为自变量,考察MSPD萃取条件。利用Design-Expert软件构建了OPFRs平均萃取效率与3种因素之间的模型。采用CCD进行三因素五水平响应面试验,共20组。其中14组为分析因子,6组作为区域中心点,以估计误差,因素水平编码见表2。每批次试验重复进行3次,结果取平均值。响应面试验设计与分析结果见附表1。将附表1试验结果进行回归拟合,得到一个二项式回归方程:


(1) *Y=*86*.*21*-*2*.*86*A+*4*.*54*B+*1*.*88*C+*1*.*39*AB+*0*.*088*AC+*3*.*69*BC-*3*.*53*A*^2^*-*10*.*58*B*^2^*-*4*.*27*C*^2^

**表2:** 响应面试验的因素和水平

Factor	Levels				
	-2	-1	0	1	2
A(Adsorbent weight/g)	0.1	0.2	0.3	0.4	0.5
B(Eluent volume/mL)	6	8	10	12	14
C(Grinding time/min)	1	3	5	7	9

#### 2.2.2 RSM回归模型可靠性分析

为评估响应面预测结果的准确性,通过Design Expert软件对OPFRs的萃取效率回归模型进行方差分析(ANOVA),结果见表3。由表3可知,回归模型*F*值为176.93,且*p*值小于10^-4^,说明仅噪声引起的“模型*F*值”出现的可能性为0.01%,表示该模型具有显著性,同时预测模型与试验的拟合度用失拟项(lack of fit)来表示,失拟项*p*值为0.0724,说明模型的纯误差不显著。模型拟合度(*R*^2^)为0.9938,说明模型回归效果显著。

**表3:** 回归模型方差分析

Source	Sum of square	Degree of freedom	Mean square	F	P	Result
Model	3586.75	9	398.53	176.93	<0.0001	significant
A	130.53	1	130.53	57.95	<0.0001	significant
B	330.33	1	330.33	146.66	<0.0001	significant
C	56.63	1	56.63	25.14	0.0005	significant
AB	15.40	1	15.40	6.84	0.0258	significant
AC	0.061	1	0.061	0.027	0.8723	nonsignificant
BC	108.78	1	108.78	48.30	<0.0001	significant
A^2^	314.03	1	314.03	139.42	<0.0001	significant
B^2^	2816.58	1	2816.58	1250.46	<0.0001	significant
C^2^	458.77	1	458.77	203.68	<0.0001	significant
Residual	22.52	10	2.25			
Lack of fit	8.18	5	1.64	0.57	0.7240	nonsignificant
Pure error	14.35	5	2.87			
Corrected total	3609.27	19				

减少不显著的模型项可以提高模型拟合度^[27]^。预测*R*^2^=0.9758,与调整后的*R*^2^=0.9881,两者非常一致,*R*^2^越接近1,说明回归模型越能分析、预测MSPD对土壤中10种OPFRs的萃取条件。变异系数<10%,表明试验的可信度和精确度高。精密度(Adeq Precision)是有效信号与噪声的比值(48.457),大于4视为合理。因此,该拟合模型适用于对OPFRs萃取效率的预测。

MSPD对土壤中OPFRs平均萃取效率的模型预测值与试验值的分布图见附图2。由附图2a可知,预测值与试验值均分散于*y=x*附近,无异常点,表明该模型能够用来分析和预测试验值。由附图2b残差与预测值的分布图可以看出,试验残差项与模型预测值分布无序,尽可能在一条直线上,表明该模型的可靠性好。

综上分析可知,该模型能用于分析和预测基质分散固相萃取土壤中10种OPFRs的平均萃取效率。

当*P*<0.05时,表示该因素是显著的模型项;*P*>0.05时,表示模型项不显著。从表3可以看出,在这种情况下,一次项*A*、*B*、*C*均达到显著水平(*P*<0.01),其中*A*、*B*对响应值*Y*的影响较大;在二次项中,*A*^2^、*B*^2^、*C*^2^(*P*<0.0001)为极显著项,三者系数均为负值,说明方程存在极大值点;在交互项中,*AB*、*BC*达到显著水平(*P<*0*.*05), *AC*对响应值的影响不显著。综合说明各个因素对*Y*的影响并不是线性关系。

#### 2.2.3 单因素之间交互作用的分析

MSPD单一因素对OPFRs萃取效率的影响与单因素分析结果具有较好的一致性。各因素之间存在交互作用。其中吸附剂用量与洗脱剂体积(*P*<0.05)、洗脱剂体积与研磨时间(*P*<0.00001)之间的交互作用更显著。

图2显示了研磨时间处于中心点5 min时,吸附剂用量和洗脱剂体积对OPFRs平均萃取回收率的交互影响。由3D响应曲面图和2D等高线响应图可以直观地看出,在同一吸附剂用量下,OPFRs平均萃取回收率随着洗脱剂体积的增加呈先上升后下降的趋势;在同一洗脱剂下,OPFRs平均萃取回收率随着吸附剂用量的增加先增加后减少。

**图2:**
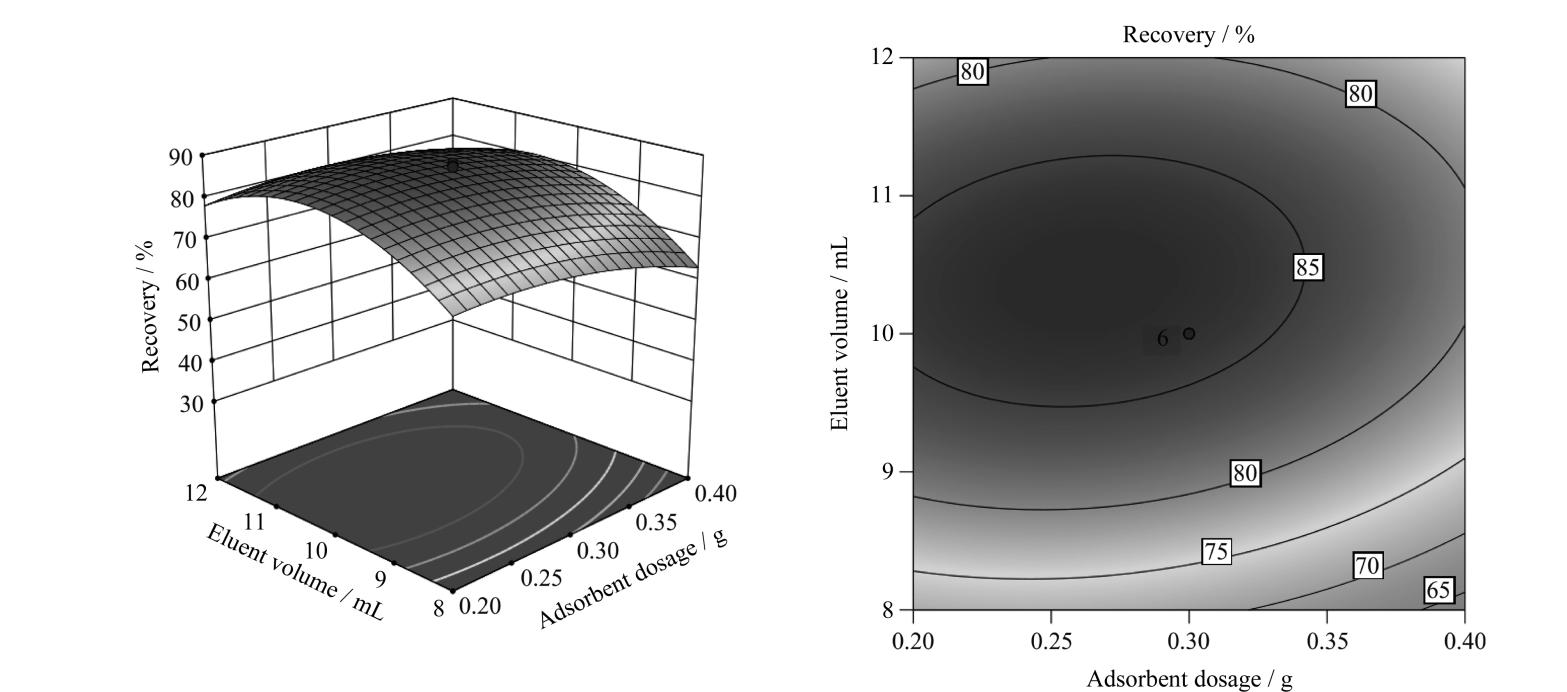
吸附剂用量与洗脱剂体积对OPFRs平均萃取效率交互影响的响应面和等高线图

图3显示了当洗脱剂体积处于中心点10 mL时,吸附剂用量和研磨时间对OPFRs萃取效率的交互作用。由图3可知,在同一吸附剂用量下,OPFRs平均萃取回收率随着研磨时间的延长先增加后减少;在同一研磨时间下,OPFRs平均萃取回收率随着吸附剂用量的增加先升高后下降。

**图3:**
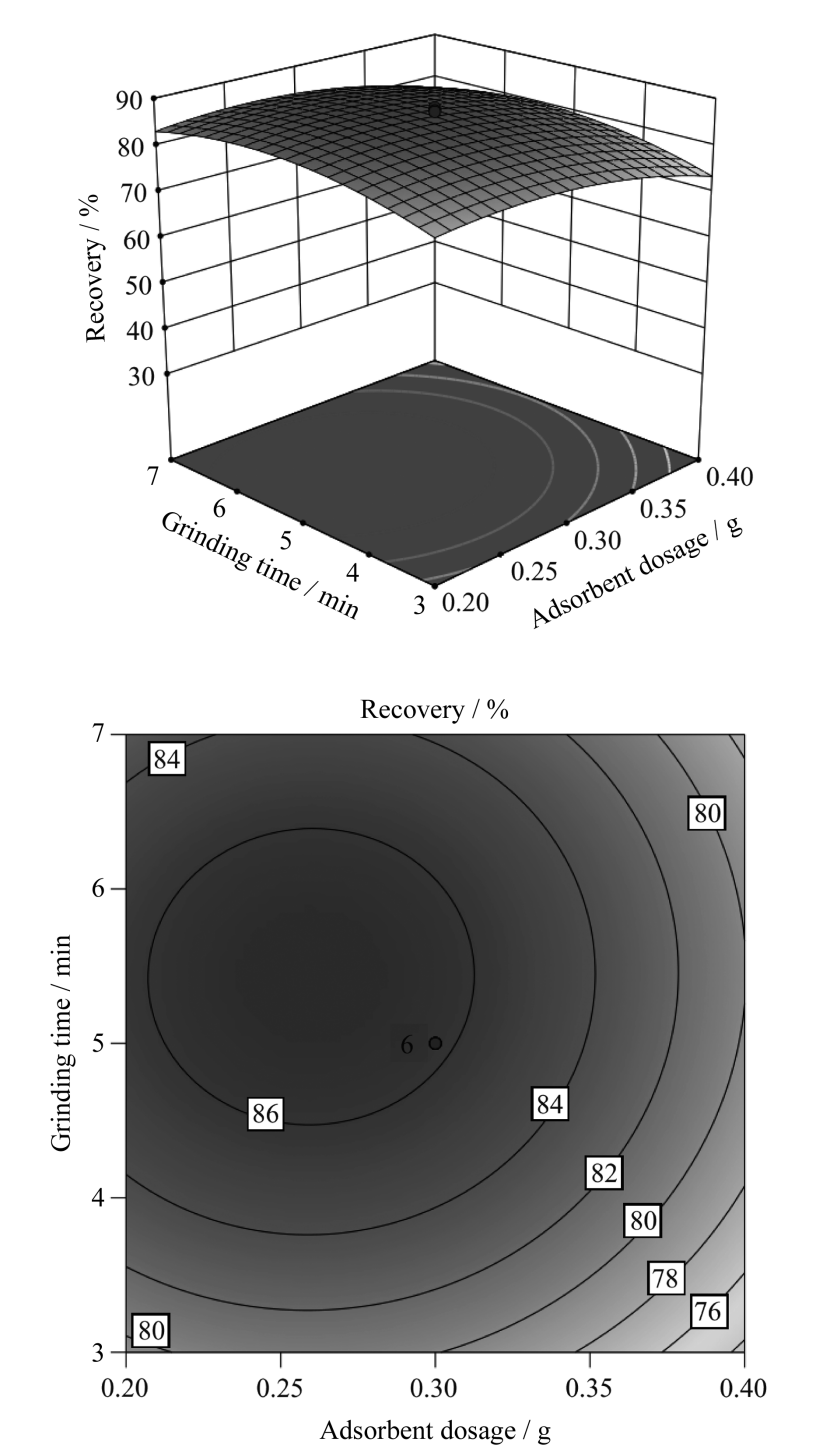
吸附剂用量与研磨时间对OPFRs平均萃取效率交互影响的响应面和等高线图

但二者交互作用相对较弱(*P*>0.05),这可能是因为即使增加了吸附剂的用量,仍需要一定的研磨时间使吸附剂与土壤中OPFRs充分接触,以获得良好的吸附效果,从而提高OPFRs萃取效率。吸附剂用量处于中心点0.3 mg时,洗脱剂体积和研磨时间对响应值*Y*的交互影响较为显著。由图4可知,当洗脱体积一定时,OPFRs平均萃取回收率随着研磨时间的增加呈现先增大后减小的变化趋势,研磨时间为5 min时,OPFRs萃取回收率最大,当研磨时间一定时,OPFRs萃取回收率随着洗脱剂体积增加呈先升高后下降的变化特征。

**图4:**
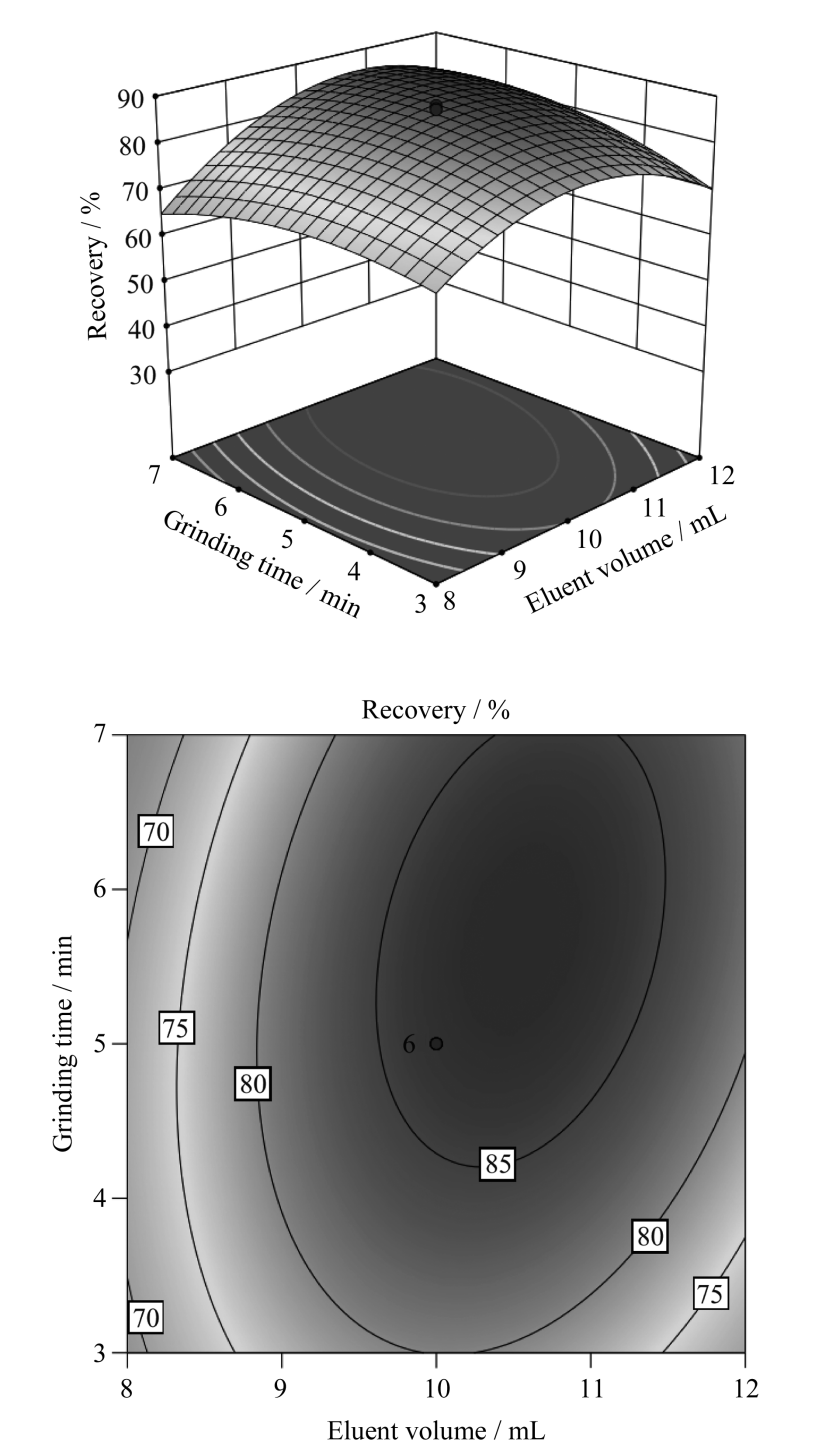
洗脱剂体积与研磨时间对OPFRs萃取效率交互影响的响应面和等高线图

综合分析发现,不同MSPD萃取条件位于中心点时,MSPD技术提取土壤中OPFRs效率最高。根据预测模型得到最优结果:0.3 g吸附剂、10 mL洗脱剂和5 min研磨时间,此时模型预测MSPD对土壤中OPFRs的平均萃取回收率达到87.5%。

### 2.3 方法学考察

#### 2.3.1 线性关系、检出限与定量限

利用10 mg/L的10种OPFRs混合标准溶液配制成质量浓度分别为1.0、10.0、50.0、100.0、500.0、1000.0 μg/L的系列标准工作溶液,以10 ng^13^C-PCB208为内标化合物,以标准工作溶液中OPFRs的质量浓度(*x*, μg/L)为横坐标,OPFRs的峰面积与内标峰面积之比(*y*)为纵坐标,建立10种OPFRs的标准工作曲线。如表4所示,10种OPFRs在1.0~1000.0 μg/L范围内具有良好的线性关系,相关系数(*r*)≥0.9982。土壤质量固定为0.2 g,以适量低浓度OPFRs加标土壤样品进行MSPD处理,分析OPFRs的峰面积响应信号,分别以3倍和10倍信噪比(*S/N*)确定方法的检出限(LOD)和定量限(LOQ)。同时用空白程序验证实验过程试剂、容器等是否污染。由于TBP、TiBP和TPrP常作为增塑剂,实验过程中使用的过滤膜、吸管等可能含有这些OPFRs,导致空白程序中有少量的TBP、TiBP和TPrP检出,这3种化合物的检出限以空白均值加3倍标准偏差来计算。结果见表4。10种OPFRs的LOD和LOQ分别为0.006~0.161 ng/g和0.020~0.531 ng/g。

**表4:** 10种OPFRs的线性方程、相关系数、检出限及定量限

Compound	Linear equation	r	LOD/ (ng/g)	LOQ/ (ng/g)
TPrP	y=4.34×10^-2^x+1.80×10^-1^	0.9988	0.072	0.240
TiBP	y=1.01×10^-2^x+1.02×10^-1^	0.9991	0.057	0.191
TBP	y=3.21×10^-2^x-1.40	0.9986	0.061	0.203
TCEP	y=4.00×10^-4^x+7.86×10^-2^	0.9990	0.052	0.173
TCIPP	y=4.70×10^-3^x-5.49×10^-2^	0.9983	0.013	0.043
TDCPP	y=1.09×10^-2^x-5.75×10^-1^	0.9986	0.025	0.083
TPHP	y=8.80×10^-3^x-1.03	0.9985	0.047	0.155
EHDPP	y=3.60×10^-3^x-8.27×10^-2^	0.9982	0.040	0.132
TPPO	y=3.10×10^-3^x-1.15×10^-1^	0.9982	0.006	0.020
TCP	y=1.00×10^-4^x-8.00×10^-4^	0.9986	0.161	0.531

*y*: the ratio of peak areas of analyte to internal standard; *x*: mass concentration, μg/L.

#### 2.3.2 回收率与精密度

以空白加标、基质加标回收率试验来考察MSPD提取土壤中OPFRs方法的性能。具体以无水硫酸钠替代土壤基质作为空白样品,向空白样品和4种实际土壤中分别添加3个水平的OPFRs混合标准溶液,使土壤中OPFRs含量分别为10、20和100 ng/g,并混合均匀静置7 d。同时MSPD过程开始前,在空白样品和实际土壤样品中添加20 ng/g的替代物D_27_-TBP,以替代物回收率来验证MSPD方法对每个样品的提取效率。空白、实际土壤中10种OPFRs和替代物的加标回收率及相对标准偏差(RSD,*n*=3)见表5。10种OPFRs的平均回收率为70.4%~115.4%, RSD为0.7%~6.7%,实际土壤中替代物D_27_-TBP的平均回收率为88.2%~95.6%。方法满足测试要求。

**表5:** 10种OPFRs和替代物在3个水平下的加标回收率及RSD(*n*=3)

Compound	Added/ (ng/g)	Blank			Electronics factory			Auto-repair plant			Paddy field			School field		
		Recovery/%	RSD/%		Recovery/%	RSD/%		Recovery/%	RSD/%		Recovery/%	RSD/%		Recovery/%		RSD/%
TPrP	10	87.1	2.3		76.0	2.0		70.4	4.7		71.9	4.4		73.2		4.7
	20	92.8	2.5		81.8	2.8		83.6	2.3		71.5	5.2		78.5		2.3
	100	95.7	3.6		77.8	3.0		75.1	1.0		103.1	1.9		88.0		3.5
TiBP	10	90.2	2.5		89.8	3.1		81.3	2.9		89.9	3.9		85.6		3.5
	20	93.5	2.9		84.1	3.4		96.8	2.0		91.9	3.6		83.2		2.2
	100	95.8	3.4		90.9	3.7		83.5	2.9		96.3	2.6		94.2		2.0
TBP	10	98.4	3.5		89.1	4.4		85.1	3.0		89.3	5.5		82.7		1.8
	20	99.5	3.9		91.4	2.6		106.9	2.6		85.0	2.9		94.8		4.2
	100	101.3	3.2		95.6	3.0		96.5	2.0		112.7	1.6		107.5		2.5
TCEP	10	92.5	3.8		79.0	1.8		83.8	6.4		87.9	5.3		84.4		1.2
	20	97.4	2.5		80.2	0.7		79.2	3.5		73.6	2.9		97.3		1.4
	100	94.8	2.0		81.1	2.4		93.9	1.1		79.3	4.2		87.5		5.7
TCIPP	10	88.8	4.4		95.7	2.6		96.2	3.7		89.7	2.8		100.0		2.1
	20	92.6	4.8		86.9	1.0		103.6	3.1		90.5	4.4		100.4		1.1
	100	95.8	3.6		93.4	2.9		97.5	3.7		96.9	6.7		92.1		2.3
TDCPP	10	99.5	3.5		81.0	1.4		70.8	1.6		88.9	1.6		79.6		4.7
	20	99.1	2.5		90.2	1.9		85.5	2.8		97.7	2.6		74.4		3.1
	100	98.4	2.0		90.1	3.5		91.9	3.9		95.3	1.8		99.4		1.4
TPHP	10	88.5	2.8		75.9	3.0		85.7	5.5		84.7	3.6		101.1		2.9
	20	95.3	3.5		81.0	4.4		109.0	4.2		85.1	2.4		101.8		2.3
	100	97.1	2.7		84.6	4.8		106.0	2.8		92.1	1.9		109.6		2.9
EHDPP	10	80.8	1.7		89.7	1.2		104.1	1.8		93.8	4.6		88.2		2.8
	20	89.5	2.1		80.9	5.0		93.0	1.4		87.7	1.9		100.3		1.6
	100	95.7	2.5		103.8	2.3		117.1	1.6		79.8	6.5		104.2		1.7
TPPO	10	95.4	3.6		93.0	3.8		90.4	1.3		95.7	2.5		105.8		4.2
	20	99.7	2.8		94.8	5.8		97.6	3.0		102.3	1.1		98.6		2.3
	100	95.5	3.1		99.5	4.3		104.2	3.7		115.4	2.5		94.7		2.0
TCP	10	80.5	3.3		75.9	3.4		78.3	3.1		78.5	4.1		78.7		4.2
	20	85.7	3.6		82.2	3.7		80.1	1.3		82.7	4.1		77.9		1.9
	100	90.4	2.8		89.8	5.0		80.4	4.6		78.3	3.4		85.2		2.8
D_27_-TBP	20	97.5	1.4		90.5	3.6		95.6	3.8		88.2	3.1		91.4		2.6

#### 2.3.3 MSPD与ASE对比

为了验证该方法的准确性,与传统的加速溶剂萃取法进行比较。同样准确称取0.2 g加标农田土壤样品(OPFRs含量为100 ng/g),以乙酸乙酯为萃取剂,萃取温度为110 ℃,压力为10^4^ kPa,循环4次,每次5 min,萃取收集液氮吹至尽干,后续与MSPD步骤相同。结果见附表2,MSPD萃取土壤中10种OPFRs的效率与ASE方法的结果基本一致,但MSPD具有操作简单、前处理耗时短和有机溶剂用量少等优点。

### 2.4 实际样品检测

利用该方法分析了苏州市不同地区(某电子厂、某汽修厂、某稻田、某校园)土壤中10种OPFRs,含量范围见表6。空白和各个实际土壤样品中10种OPFRs的色谱图见图5。在4个实际土壤样品中发现10种OPFRs的总含量范围为6.49~26.00 ng/g。电子厂和汽修厂土壤中OPFRs含量显著高于农田和校园,说明电子厂和汽修厂存在较高的OPFRs污染来源,工厂土壤中主要污染物为TCIPP、TPPO、TCEP和TDCPP,它们在电子厂土壤中含量分别为5.30、4.44、4.54、4.20 ng/g,在汽修厂土壤中含量分别2.70、3.93、7.60、5.04 ng/g。TPPO主要用于合成中间体、催化剂和萃取剂等,目前关于土壤中TPPO污染的报道较少,仅有辽河上游河岸带土壤中发现TPPO为主要污染物的报道^[28]^,而农田土中主要污染物为TPrP、TDCIP和TiBP。这与已报道的北京地区农田土以TCIPP((3.36±5.61) ng/g)为主的结论并不一致^[29]^。校园土中主要污染物以TCIPP和TPrP为主。苏州地区土壤中OPFRs检出含量远低于尼泊尔市(25~27900 ng/g)^[30]^。总体上目前苏州地区土壤中OPFRs污染水平较低。

**表6:** 不同采样点土壤中10种有机磷阻燃剂的含量

Compound	Electronics factory	Auto-repair factory	Paddy field	School field
TPrP	1.82	2.53	2.21	1.75
TiBP	2.72	0.29	0.92	0.62
TBP	2.26	0.24	0.51	0.14
TCEP	4.54	7.60	0.24	ND
TCIPP	5.30	2.70	0.22	4.14
TDCPP	4.20	5.04	1.52	ND
TPHP	0.24	ND	ND	0.65
EHDPP	ND	0.41	ND	ND
TPPO	4.44	3.93	0.33	0.20
TCP	0.49	0.42	0.54	ND
∑OPFRs	26.00	23.15	6.49	7.50

ND: not detected; ∑OPFRs: the total content of the 10 OPFRs.

**图5:**
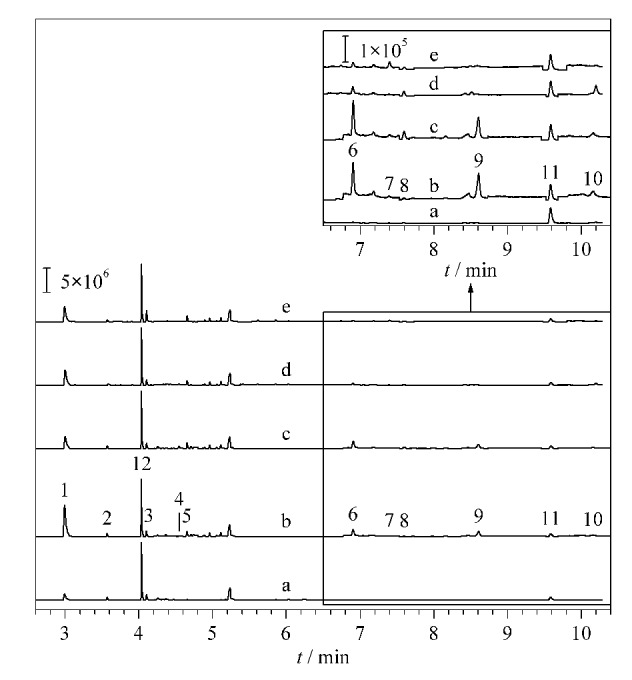
空白和实际土壤样品中10种OPFRs的色谱图

## 3 结论

本文建立了一种MSPD-GC-MS/MS检测土壤中10种OPFRs的方法。单因素分析法结合RSM系统地分析了基质固相分散萃取土壤中OPFRs回收率的关键影响因素以及因素之间的相互作用,建立了OPFRs萃取回收率为响应值的回归模型,模型预测值与试验值具有良好的拟合度,确定了最优的萃取条件。该方法涉及实验少、有机溶剂用量少、操作步骤简单便捷,且具有良好的准确度和精密度。将该方法应用于不同功能区土壤中OPFRs残留量的监测分析,发现了电子厂和汽修厂土壤中高水平TPPO污染,后续有必要开展这类污染物的生态环境风险评估。
